# Development and Evaluation of a Rapid and Sensitive EBOV-RPA Test for Rapid Diagnosis of Ebola Virus Disease

**DOI:** 10.1038/srep26943

**Published:** 2016-06-01

**Authors:** Mingjuan Yang, Yuehua Ke, Xuesong Wang, Hang Ren, Wei Liu, Huijun Lu, Wenyi Zhang, Shiwei Liu, Guohui Chang, Shuguang Tian, Lihua Wang, Liuyu Huang, Chao Liu, Ruifu Yang, Zeliang Chen

**Affiliations:** 1 Institute of Disease Control and Prevention, Academy of Military Medical Sciences, 100071, Beijing, China; 2 China Mobile Laboratory Response Team for Ebola in Sierra Leone, Freetown, Sierra Leone; 3 Key Laboratory of Jilin Province for Zoonosis Prevention and Control, Changchun 130122, China; 4 Wangjing Hospital, China Academy of Traditional Chinese Medicine, 100102, Beijing, China; 5 Institute for Viral Disease Control and Prevention, Chinese Center for Disease Control and Prevention, Beijing 102206, China; 6 State Key Laboratory of Pathogen and Biosecurity, 100071, Beijing, China; 7 Beijing Key Laboratory of POCT for Bioemergency and Clinics, 100071, Beijing, China; 8 Key Laboratory of Zoonosis of Liaoning Province, College of Animal Science and Veterinary Medicine, Shenyang Agriculture University, 110866, Shenyang, China

## Abstract

Confirming Ebola virus disease (EVD), a deadly infectious disease, requires real-time RT-PCR, which takes up to a few hours to yield results. Therefore, a rapid diagnostic assay is imperative for EVD diagnosis. A rapid nucleic acid test based on recombinase polymerase amplification (EBOV-RPA) was developed to specifically detect the 2014 outbreak strains. The EBOV-RPA assay was evaluated by testing samples from suspected EVD patients in parallel with RT-PCR. An EBOV-RPA, which could be completed in 20 min, was successfully developed. Of 271 patients who tested positive for Ebola virus by RT-PCR, 264 (sensitivity: 97%, 95% CI: 95.5–99.3%) were positive by EBOV-RPA; 101 of 104 patients (specificity: 97%, 95% CI: 93.9–100%) who tested negative by RT-PCR were also negative by EBOV-RPA. The sensitivity values for samples with a Ct value of <34, which accounted for 95.59% of the samples, was 100%. Discordant samples positive by RT-PCR but negative by EBOV-RPA had significantly high Ct values. Results of external quality assessment samples with EBOV-RPA were 100%, consistent with those of RT-PCR. The EBOV-RPA assay showed 97% sensitivity and 97% specificity for all EVD samples tested, making it a rapid and sensitive test for EVD diagnosis.

The ongoing Ebola virus disease (EVD) outbreak in West Africa is the largest since its first discovery in 1976^1^,^2^,^3^. As of September 16, 2015, the World Health Organization reported 28,220 confirmed, probable, and suspected EVD cases and 11,291 deaths in Guinea, Liberia, and Sierra Leone^4^. EVD is an infectious disease characterized by high fatality and rapid progress to an outcome of death or recovery, with a fatality rate of 50–90%^5^,^6^,^7^. For the ongoing outbreak, the fatality rate for confirmed cases (15,199) is 74.3%. Due to the rapid progression to death and the long time required to obtain laboratory results, rapid diagnosis is extremely important for timely triage and treatment^8^.

EVD patients normally present with non-specific symptoms, including fever, headache, and vomiting. The symptoms of EVD overlap with those of other diseases prevalent in West Africa, which complicates the clinical diagnosis, management, and treatment of EVD patients. EVD is confirmed by laboratory diagnosis, with real-time RT-PCR being the most commonly used assay. The diagnosis of EVD in West Africa involves the collection of blood and/or swab samples, transportation to the field laboratory, and testing by real-time RT-PCR. The entire process from sample collection to obtaining results takes several hours^9^.

Suspected EVD patients are triaged at holding centers, while confirmed patients are transferred to treatment centers and EVD-negative individuals are discharged after 2 sequential negative tests. At holding centers, samples from suspected patients are collected and sent to the laboratory for diagnosis confirmation. The run time for the laboratory diagnosis ranges from several hours to several days, which is a long time, considering the rapid progression of EVD. A prolonged stay at a holding center also increases the exposure risk to Ebola virus^10^.

The dipstick test is the most widely used test for point-of-care (POC) diagnosis. This test does not require an external instrument and can be used by technicians with minimal extra training. Broadhurst *et al.* recently published an evaluation study of the dipstick test for EVD diagnosis^11^. Despite positive predictive values (PPVs) of 100%, the sensitivity of the dipstick test is limited to samples with cycle threshold values <26.

Considering this situation, the development of a rapid test with high sensitivity is urgently needed. Here, we report the evaluation of a new nucleic acid (NA)-based test, EBOV-RPA, for rapid and sensitive EVD diagnosis. The EBOV-RPA is based on recombinase polymerase amplification (RPA) technology^12^,^13^, which is performed at a constant temperature and has a run time shorter than 15 min. The aim of the study was to assess the test performance of the EBOV-RPA assay.

## Results

Of all available, common rapid NA-based methods evaluated, the RPA assay—one of the most rapid and specific, was chosen as the detection method^14^,^15^. An EBOV-RPA assay that specifically detects strains circulating in the 2014 outbreak was successfully developed. The RPA assay consistently detected as low as 10 copies per reaction (Fig. S1). A simplified sample treatment procedure that avoids NA extraction was developed (Table S2). With the simplified procedure, the EBOV-RPA assay can be completed in 20 minutes.

**Figure 1:**
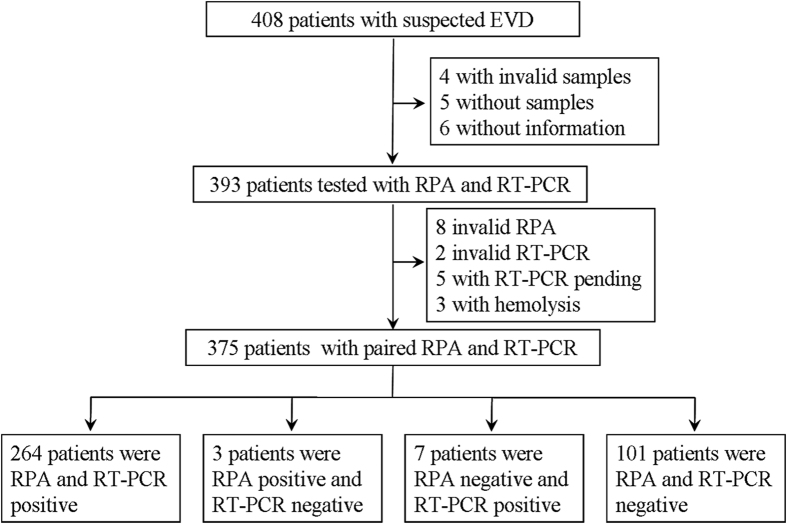
Overview of the EBOV-RPA evaluation study.

The performance evaluation study was carried out in Sierra Leone (Fig. 1). We enrolled 408 patients, of whom 15 were subsequently excluded, leaving 393 to be tested by EBOV-RPA and RT-PCR. Of these 393 patients, 8 were invalid by EBOV-RPA, 2 were invalid by RT-PCR, 5 pending by RT-PCR, and 3 had serious hemolysis; the remaining 375 patients showing valid RPA and RT-PCR results were included in the final analysis (Fig. 1). Of the 361 suspected EVD patients with available demographic information, 241 (66.8%) were from the Western Area Urban District of Sierra Leone, 109 (30.2%) were from the West Area Rural District, and 11 (3.0%) were from other districts (Table 1). The ages of these suspected patients ranged from 0 to 96 years (mean: 30.6, 95% CI: 26.9–34.3), and 166 (52.8%) of them were female. The median symptom onset time after specimen collection was 4.66 days (range: 0–16, 95% CI: 3.86–5.45) in the 248 patients with available data. Of these patients, 278 have recorded symptom information. The most prevalent symptoms included weakness (86.8%), loss of appetite (84.4%), fever (82.2%) and muscle pain (69.0%). A significant proportion of these suspected EVD patients had multiple symptoms: 130 (46.8%) of them had 6–10 symptoms, and 76 (27.3%) had >11 symptoms. Of these patient samples, 144 (58.15%) were collected in <3 days, 83 (33.5%) were collected in 4–7 days, and 21 (8.5%) were collected in over 7 days.

After excluding invalid samples or test results, 375 samples were included in the final evaluation analysis. The overall sensitivity was 97% (95% CI: 95.5–99.3) and the specificity was 97% (95% CI: 93.9–100; Table 2). For blood samples, the sensitivity was 97% (95% CI: 95.1–99.4) and the specificity was 97% (95% CI: 93.1–100), and for swab samples, the sensitivity was 98% (95% CI: 94.3–100) and the specificity was 97% (95% CI: 91.6–100). We next analyzed the sensitivity and specificity of the RPA assay for samples with different Ct values. For 255 samples (96.59%) with Ct values <34, the sensitivity for both sample types was 100%. For the 10 samples (3.79%) with Ct values of 34–36, the overall sensitivity was 70%, and the sensitivity was 66.7% for blood samples and 100% for swabs. For 6 samples (2.27%) with Ct values >36, the sensitivity was 33.3% for blood samples and 0% for the only swab sample. For all the 3 Ct ranges, the PPV was 100% (Table 3). ROC analysis showed that the EBOV-RPA assay is highly sensitive and specific; the AUC was 0.9652 (Fig. S4).

The Ct value distribution of these tested samples was analyzed, with 134 samples (49.45%) having Ct values <26, 121 (44.65%) with Ct values in range of 26–34, and only 16 (5.9%) with Ct values >34 (Table S3). Viral loads were calculated from Ct values using a standard curve. The viral loads of the tested samples ranged from 10^3^ to 10^10 ^copies/mL. The viral loads of swabs were lower than that of the blood samples (Fig. S2). The mean of the threshold time of EBOV-RPA was significantly shorter than that of RT-PCR (Table S3; 5.53 vs. 37.78 minutes, F = 225.401, p < 0.001), indicating that EBOV-RPA amplification is more rapid than RT-PCR (Fig. S3).

The RPA assay was also used to detect the blinded samples for external quality assessment (EQA), which was sponsored by the WHO. Of the 10 blinded samples, 5 had inactivated EBOV, while the other 5 had EBOV RNA. The EBOV-RPA results were completely consistent with those of RT-PCR (Table S5), and samples with low Ct values in RT-PCR also showed low Ct values by EBOV-RPA. The RPA assay yielded results in 30 min, while RT-PCR yielded results approximately 2 h later. According to the assessment result, our EQA tests were 100% correct and the EBOV-RPA assay was more rapid.

Compared with RT-PCR, EBOV-RPA generated discordant results for a few samples. RNA from the discordant samples positive by RT-PCR, but negative by EBOV-RPA, was re-tested by EBOV-RPA. Of these samples, 1 was positive and the other 6 were still negative. The Ct values of all 5 samples were >34. The discordant samples were also tested with another RT-PCR kit (Ebov-NP) targeting the NP gene. All 10 samples positive by Ebov-GP were also positive by Ebov-NP; however, the 2 samples positive by EBOV-RPA but negative by Ebov-GP were also positive by Ebov-NP (Table 4). This indicated that the 2 samples in question were not false positives.

## Discussion

The time from symptom onset to outcome in EVD is <10 days on average^6^. However, because of the time required for sampling, transportation, laboratory diagnosis, and obtaining results, the diagnosis and timely treatment of suspected EVD patients is delayed, increasing the risk of exposure to other persons^16^,^17^. Mara *et al.* recently reported field validation of the dipstick immunoassay for EVD. Although a PPV of 100% was reported, the test was only applicable for samples with high virus load^11^. Indeed, there is a significant proportion of clinical samples with low virus load. The dipstick assay cannot accurately define these samples as negatives. Alternatively, the samples need to be further defined by RT-PCR in a reference laboratory. Therefore, there is a need for a rapid test that can detect EBOV accurately. In the current outbreak, laboratory testing with real-time PCR is widely used in the affected areas. However, the requirements of sophisticated thermocycler and complex sample treatment procedures have limited its applications in point-of-care testing^18^. Recombinase polymerase amplification (RPA) overcomes the technical difficulties posed by current amplification methods. It does not require thermal denaturation of template and operates at a low and constant temperature, without reliance on expensive thermocycler. In combination with a novel fluorescent probe, it can be monitored with a portable, real-time fluorometer, with portable battery pack. RPA has also been shown to be highly resistant to crude samples in comparison to PCR, suggesting applications in on-the-spot field testing with crude nucleic acid extraction^19^.

Using RPA technology, we developed and evaluated a new assay that detects the EBOV NA gene. The principle of RPA is similar to that of RT-PCR but is performed under isothermal conditions. Thus, the amplification time is <15 min, or approximately one-fourth that of the most rapid RT-PCR assay. Furthermore, the sensitivity of the RPA assay is comparable to that of RT-PCR. The RPA assay has been successfully used to detect bioterrorism pathogens and many other microorganisms^15^,^20^,^21^. By analyzing the genome sequences of the 2014 outbreak strains, we identified a conserved genomic region as a signature sequence for development of the EBOV-RPA assay, which ensured that the assay could more specifically detect the currently circulating strains. After optimization, the assay could detect as few as 10 copies per reaction. Because NA extraction is time consuming, a simplified sample treatment that avoided the extraction step without compromising sensitivity was successfully developed. This simplified procedure renders the RPA assay feasible for rapid diagnosis.

Sensitivity is essential for a rapid test. The overall sensitivity, specificity, PPV, and NPV of the EBOV-RPA assay for the panel of 375 samples demonstrated suitable performance of the assay for rapid EVD diagnosis. Most importantly, the PPV for all tested samples was 100%, i.e., the samples positive by EBOV-RPA were true positive samples. For samples with Ct values <34 (representing 94.1% of the samples), the sensitivity was 100% for both sample types. Therefore, EBOV-RPA is very accurate for EVD diagnosis. We further analyzed the discordant samples positive by RT-PCR but negative by EBOV-RPA. All 6 false-negative samples identified by EBOV-RPA had Ct values >35, 4 of which were in the gray zone (Ct = 36–40) of the Ebov-GP kit. Compared with RT-PCR, EBOV-RPA generated 3 false positives according to the Ebov-GP kit. However, further confirmation by the Ebov-NP kit showed that 2 of them were actually true positives. This in consistence might be that both EBOV-RPA and Ebov-NP target NP genes of Ebola virus. These results also implied that for the clinical diagnosis of EVD, a 2-target strategy would improve the diagnostic accuracy.

Although the RPA assay was not compared directly with the dipstick assay, both assays were compared with RT-PCR. We evaluated the advantage of RPA by putatively compared with dipstick assay. The RPA assay offers several advantages over the dipstick assay. Firstly, the RPA assay is specific, sensitive and easy to develop over a short period of time. As with real-time PCR, the RPA assay used a primer pair and a probe highly specific for the target sequence^22^. RPA can detect samples with Ct values up to 32 with 100% sensitivity and is thus 100-fold more sensitive than the dipstick method. A possible reason for the enhanced sensitivity is that the RPA assay targets the NA gene, while the dipstick assay targets an antigen. Secondly, RPA assay results could be quantitatively generated in the form of virus titer levels. For the dipstick assay, the operator reads the result and it can be difficult to define whether a given sample is positive or negative. Lastly, the short run time of the RPA assay is the main advantage over the dipstick assay. While dipstick assay can be used with minimal training and does not require power to get a result, RPA assay can be performed without nucleic acid extraction and monitored by portable fluorometer with battery. These characteristics make RPA of great importance for rapid diagnosis of EVD.

The present study has a limitation. The evaluation study was not performed under real POC conditions, such as Ebola holding centers or treatment centers. However, we performed the tests under environments simulating the POC conditions. We think that the sensitivity and specificity of the RPA assay should not have been affected by the operating site, despite this limitation.

In summary, we evaluated a rapid NA test for rapid diagnosis of EVD. The superior performance of the EBOV-RPA and its comparable performance to RT-PCR indicate that it is also appropriate for laboratory diagnosis. The RPA assay thus shows great potential for POC testing of infectious diseases, particularly emerging infectious diseases.

## Methods

### Ethics statement

This study was conducted as part of the surveillance and public health response to contain the 2014 EVD outbreak in Sierra Leone. Samples were collected for EVD testing and outbreak surveillance during the EVD outbreak under an agreement between the Sierra Leone and Chinese governments. Treatment was conducted in accordance with the protocols for viral hemorrhagic fever under the urgent interim guidance for case management established by the WHO. The protocol for this study was approved by the Ethics Committee of the Department of Health in Freetown. Written informed consent was given to every patient enrolled prior to the start of this study.

### Development of the EBOV-RPA assay

A pair of primers and 1 probe were designed to specifically detect Ebola Zaire viruses including circulating strains from the 2014 EBOV outbreak (Table S1). The sequence spanning the amplification region was synthesized and cloned into the pSQ380-MS2 plasmid to generate pSQMS2-NP. Armored RNA was prepared from pSQMS2-NP. The RT-RPA assay was performed in a 50-μL volume using the TwistAmp^TM^ Exo Kit (TwistDx, Cambridge, UK), 420 nM RPA primer F and R, 120 nM exo-probe, and 14 mM magnesium acetate. All reagents except for the template or sample RNA and magnesium acetate were prepared in a master mix, which was aliquoted into each tube of a 0.2-mL 8-tube strip containing a dried enzyme pellet^13^. Magnesium acetate was pipetted into the tube lids. Subsequently, 2 μL sample was added to the tubes. The lids were closed, and the magnesium acetate was centrifuged into the tubes using a mini-spin centrifuge. The tubes were immediately used for amplification and detection. Ten-fold serial dilutions of armored RNA standard ranging from 10^8^ to 10^1 ^molecules/μL were tested by the EBOV-RPA assay in 8 replicates. The Ct number was plotted against the number of molecules in each tube comprising the standard curve. The lowest concentration of the dilution that could be detected was determined as the detection limit.

### EBOV-RPA and RT-PCR reactions

The prepared RPA assay tubes were run on a Tube Scanner device (Qiagen Lake Constance, Stockach, Germany), Mini-8 real-time PCR system (Coyote, Beijing, China), or LightCycler LC96 Real-Time PCR System (Roche, Switzerland). Fluorescence was monitored at 42 °C for 15 min. For the Tube Scanner, the reaction temperature was set at 42 °C. The reaction conditions included a 1-min pre-incubation, followed by fluorescence measurement at intervals of 20 s for 14 min. The results obtained with the Tube Scanner were used to define the threshold time, at which point the fluorescence intensity increased exponentially. For the real-time PCR systems, a real-time PCR-like protocol was used: 1 min of pre-incubation at 42 °C, followed by 30 cycles of 42 °C 10 s and 42 °C for 10 s (fluorescence detection in channel of FAM). The RPA results obtained on the real-time PCR systems were defined in terms of Ct values. The RT-PCR assays, including those obtained with the Ebov-GP and Ebov-NP kits that specifically detect GP and NP respectively, were run on a LightCycler LC96 system essentially as recommended by manufacturer (Puruikang Biotechnology Co., Ltd, Shenzhen, China)^23^. Briefly, a 25-μl reaction system contained 20 μl reaction solution A, 2 μl reaction solution B, and 3 μl RNA extracts. Cycling conditions consisted of an initial 42 °C step for 5 min and 94 °C for 10 s, followed by 40 cycles of 94 °C for 5 s, 55 °C for 30 s (florescent signal collection, FAM channel), and 25 °C for 10 s.

### Simplified sample treatment

Aliquots (10 μL) of the blood or swab samples were mixed with AVL, Trizol, and sample solution (Coyote, Beijing, China) at ratios of 2:1, 1:1, 1:2, and 1:4. The mixtures were heat-treated at 98 °C for 3 min and then centrifuged at 3000 × *g* for 1 min. The supernatants were diluted 2, 4, 8, 16, and 32-fold and then used in the EBOV-RPA test. The sample treatment resulting in the shortest threshold time was chosen as the simplified sample treatment method.

### Evaluation procedure

The EBOV-RPA assay evaluation study was performed at the China-CDC JUI laboratory at Freetown, Sierra Leone. An overview of the evaluation study is depicted in Fig. 1. Sample aliquots were subjected to viral RNA extraction or simplified treatment of heat denaturation. The extracted RNA or treated samples were studied by EBOV-RPA or RT-PCR assays by different staff members. Two other staff members blinded to the assays independently recorded the results, which were not shared with clinicians or used for medical management. RT-PCR results were subsequently recorded. All staff participating in the study followed strict biosafety precautions according to WHO guidelines.

Whole blood or swab samples from suspected EVD patients were sent to the JUI laboratory (JUI lab) for Ebola testing. Several batches of samples were sent to JUI lab for testing each day. Samples received before 1:00 PM were selected for the evaluation study so that the samples could be treated and tested on the same day. Samples from suspected EVD patients who completed a questionnaire form were included. Samples were excluded when not correctly stored, transported, or stored for >48 hours after sampling. Once received, the samples were brought into a BSL-3 mobile laboratory for sample treatment, and sample aliquots were subjected to RNA extraction with QIAamp Viral RNA Mini Kit (Qiagen, Germantown, MD, USA) or simplified treatment as described above. Extracted RNAs were detected by RT-PCR in a BSL-2 laboratory, and simplified treated samples were detected by EBOV-RPA in another simulated point-of-care zone.

### Statistical analysis

The test sensitivity is the proportion of all RT-PCR-positive samples that are positive by EBOV-RPA. The test specificity is the proportion of all RT-PCR-negative samples that are also negative by EBOV-RPA. Data analysis was performed using SPSS, version 17.0. Continuous variables were analyzed with one-way ANOVA and Student’s *t*-test. The chi-squared test was used for categorical independent and paired variables. The cutoff value was determined by performing receiver operating characteristic (ROC) analysis, in which the sensitivity and specificity were calculated as a function of the cutoff value. The value of 1-specificity was plotted against the sensitivity, and the areas under the ROC curves (AUCs) were calculated. p < 0.05 was considered statistically significant.

## Additional Information

**How to cite this article**: Yang, M. *et al.* Development and Evaluation of a Rapid and Sensitive EBOV-RPA Test for Rapid Diagnosis of Ebola Virus Disease. *Sci. Rep.* **6**, 26943; doi: 10.1038/srep26943 (2016).

## Supplementary Material

Supplementary Information

## Figures and Tables

**Table 1:** Demographic characteristics of enrolled suspected EVD patients.

Characteristics	No. (%) of Patients
Sex (n = 314)	
Female	166 (52.8)
Age (n = 359), Mean (range, 95% CI)	30.6 (0–96, 26.9–34.3)
≤18	101 (28.1)
18–45	193 (53.8)
>45	65 (18.1)
District of origin (n = 361)	
Western urban	241 (66.8)
Western rural	109 (30.2)
Others	11 (3.0)
Symptom onset to specimen (n = 248), mean (range, 95% CI)	
≤3	144 (58.1)
4–7	83 (33.5)
>7	21 (8.5)
Symptom	
Fever	221 (82.2)
Vomiting/nausea	177 (66.5)
Diarrhea	151 (56.8)
Weakness	230 (86.8%)
Loss of appetite	221 (84.4%)
Abdominal pain	167 (64.5%)
Chest pain	81 (60.4%)
Muscle pain	185 (69.0%)
Joint pain	178 (67.7%)
Headache	160 (61.1%)
Cough	66 (49.6%)
Difficult breathing	91 (34.9%)
Difficult swallowing	88 (34.0%)
Sore throat	54 (42.2%)
Jaundice	39 (31.7%)
Red eye	116 (45.8%)
Skin rash	37 (14.6%)
Hiccups	60 (22.6%)
Sensitive to light	34 (26.6%)
Coma	22 (16.9%)
Confused of disoriented	54 (41.9%)
Unexplained bleeding	7 (4.9%)
Multiple symptom (n = 278)	
1–5	70 (25.2)
6–10	130 (46.8)
≥11	76 (27.3)

**Table 2:** Overall performance of RPA versus RT-PCR.

Sample type	Sensitivity (%, 95% CI)	Specificity (%, 95% CI)	PPV (%)	NPV (%)	Positive LR	Negative LR
Blood and swabs (n = 375)	264/271 (97, 95.5–99.3)	101/104 (97, 93.9–100)	99	94	33.8	0.03
Blood (n = 288)	213/219 (97, 95.1–99.4)	67/69 (97, 93.1–100)	99	92	33.6	0.03
Swabs (n = 87)	51/52 (98, 94.3–100)	34/35 (97, 91.6–100)	98	97	34.3	0.02

**Table 3:** Performance of RPA in terms of sample type and different Ct value ranges.

Sample type	Ct value range		
	<34	34–36	>36
Blood and swab			
Percent (%)	96.59	3.78	2.27
Sensitivity (%)	255/255 (100)	7/10 (70)	2/6 (33.3)
PPV(%)	100	100	100
Blood			
Sensitivity (%)	205/205 (100)	6/9 (66.7)	2/5 (40.0)
PPV(%)	100	100	100
Swab			
Sensitivity (%)	50/50 (100)	1/1 (100)	0/1 (0)
PPV(%)	100	100	100

**Table 4:** Confirmation of discordant samples between the RT-PCR and EBOV-RPA assays.

Sample type	Ebov-GP	Ebov-NP	EBOV-RPA Sample	EBOV-RPA RNA
B	34	33.02	NA	15.94
B	35	33.8	NA	NA
B	35.3	34.22	NA	NA
B	36.5	35.04	NA	NA
S	36.5	35.12	NA	NA
B	37.5	36.41	NA	NA
B	38.5	37.26	NA	NA
B	NA	37.68	16.5	ND
B	NA	36.42	20.6	ND
S	NA	NA	12.8	ND

B: blood sample; S: swab sample; Ebov-GP: the kit targeting the EBOV GP gene; Ebov-NP: the kit targeting the EBOV NP gene; EBOV-RPA sample: sample detection by the EBOV-RPA assay; EBOV-RPA RNA: detection of extracted RNA; NA: not available; ND: not detected; “−” not applicable.
