# Supplementary material for: Development and Evaluation of a Rapid and Sensitive EBOV-RPA Test for Rapid Diagnosis of Ebola Virus Disease
Source: Sci Rep. 2016 Jun 1;6:26943. doi: 10.1038/srep26943 (PMC4887875; doi:10.1038/srep26943)
Supplement: Supplementary Information [file srep26943-s1.doc]

**Development and Evaluation of a Rapid and Sensitive EBOV-RPA Test for Point-of-Care Diagnosis of Ebola Virus Disease**

Mingjuan Yang^1, *^, Yuehua Ke^1,8^,^*^, Xuesong Wang^1,8, *^, Hang Ren^1, *^, Wei Liu^1,8, *^, Huijun Lu^3,8, *^, Wenyi Zhang^1,8^, Shiwei Liu^6^, Guohui Chang^1,8^, Shuguang Tian^1,8^, Lihua Wang^4,8^, Liuyu Huang^1^, Chao Liu^1,8^, Ruifu Yang^2,5,8^, Zeliang Chen^1,7,8^

^1^ Institute of Disease Control and Prevention, Academy of Military Medical Sciences, 100071, Beijing, China

^2^ State Key Laboratory of Pathogen and Biosecurity, 100071, Beijing, China

^3^ Key Laboratory of Jilin Province for Zoonosis Prevention and Control, Changchun 130122, China;

^4^ Institute for Viral Disease Control and Prevention, Chinese Center for Disease Control and Prevention, Beijing 102206, China.

^5^ Beijing Key Laboratory of POCT for Bioemergency and Clinics, 100071, Beijing, China

^6^ Wangjing Hospital, China Academy of Traditional Chinese Medicine, 100102, Beijing, China

^7^ School of Medicine, Shihezi University, 832003, Shihezi, China

^8^ China Mobile Laboratory Response Team for Ebola in Sierra Leone, Freetown, Sierra Leone

Correspondence and requests for materials should be addressed to Liuyu Huang, [huangliuyuly@163.com](mailto:huangliuyuly@163.com); Chao Liu, liuchao9588@sina.com; Ruifu Yang, ruifuyang@gmail.com; Zeliang Chen, zeliangchen@yahoo.com

^*^These authors contributed equally to this work.

Supplementary Material

Table of contents

Supplementary results [3](#__RefHeading___Toc448844800)

Sensitivity of the EBOV-RPA assay [3](#__RefHeading___Toc448844801)

Simplified sample treatment [3](#__RefHeading___Toc448844802)

Supplementary data [4](#__RefHeading___Toc448844803)

Figures [4](#__RefHeading___Toc448844804)

Figure S1 Sensitivity of the EBOV-RPA assay [4](#__RefHeading___Toc448844805)

Figure S2 Real-time PCR results showing a standard curve (Ebov-GP) and viral load distributions [5](#__RefHeading___Toc448844806)

Figure S3 Distribution of Ct values and threshold times [6](#__RefHeading___Toc448844807)

Figure S4 ROC curve of the EBOV-RPA assay for EVD [7](#__RefHeading___Toc448844808)

Tables [8](#__RefHeading___Toc448844809)

Table S1 Primers and probe used in the present study [8](#__RefHeading___Toc448844810)

Table S2 Effect of sample treatment on amplification [9](#__RefHeading___Toc448844811)

Table S3 Characteristics of CT values and threshold times observed in the RT-PCR and RPA assays [10](#__RefHeading___Toc448844812)

Table S4 Ct value distribution of the RT-PCR positive samples [11](#__RefHeading___Toc448844813)

Table S5 Detection results of blinded samples from External Quality Assessment (EQA) [12](#__RefHeading___Toc448844814)

# Supplementary results

## Sensitivity of the EBOV-RPA assay

Serial dilution of armored RNA was detected with the EBOV-RPA assay. The results showed that the RPA assay could detect as low as 10 copies per reaction (Figure S1). No cross-reactions were observed when amplifying other pathogen-derived RNAs.

## Simplified sample treatment

Samples were mixed with AVL, Trizol, or sample solution; heat denatured; and then tested by the RPA assay. As shown in Table S2, treatment with AVL and Trizol greatly inhibited the amplification, while sample solution treatment generated positive amplification under all the dilutions. Taking into account the amplification efficiency and inhibition factors, the simplified sample treatment resulted in 4-fold enhanced amplification with sample solution and denaturation at 98°C for 3 min.

# Supplementary data

## Figures

### Figure S1 Sensitivity of the EBOV-RPA assay


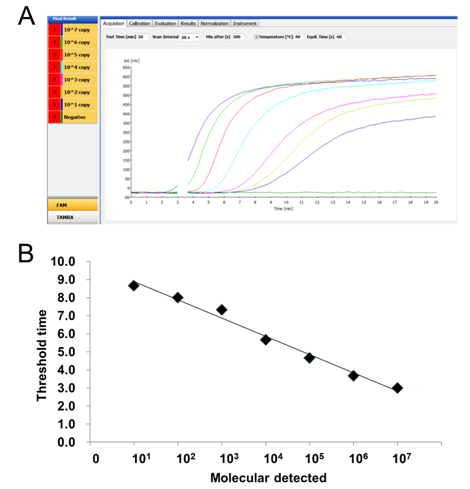


(A) Real-time fluorescence measurements using a dilution range of 10^7^–10^1^ molecules/μL of standard. (B) Analytical sensitivity determined using the standard curve.

### Figure S2 Real-time PCR results showing a standard curve (Ebov-GP) and viral load distributions


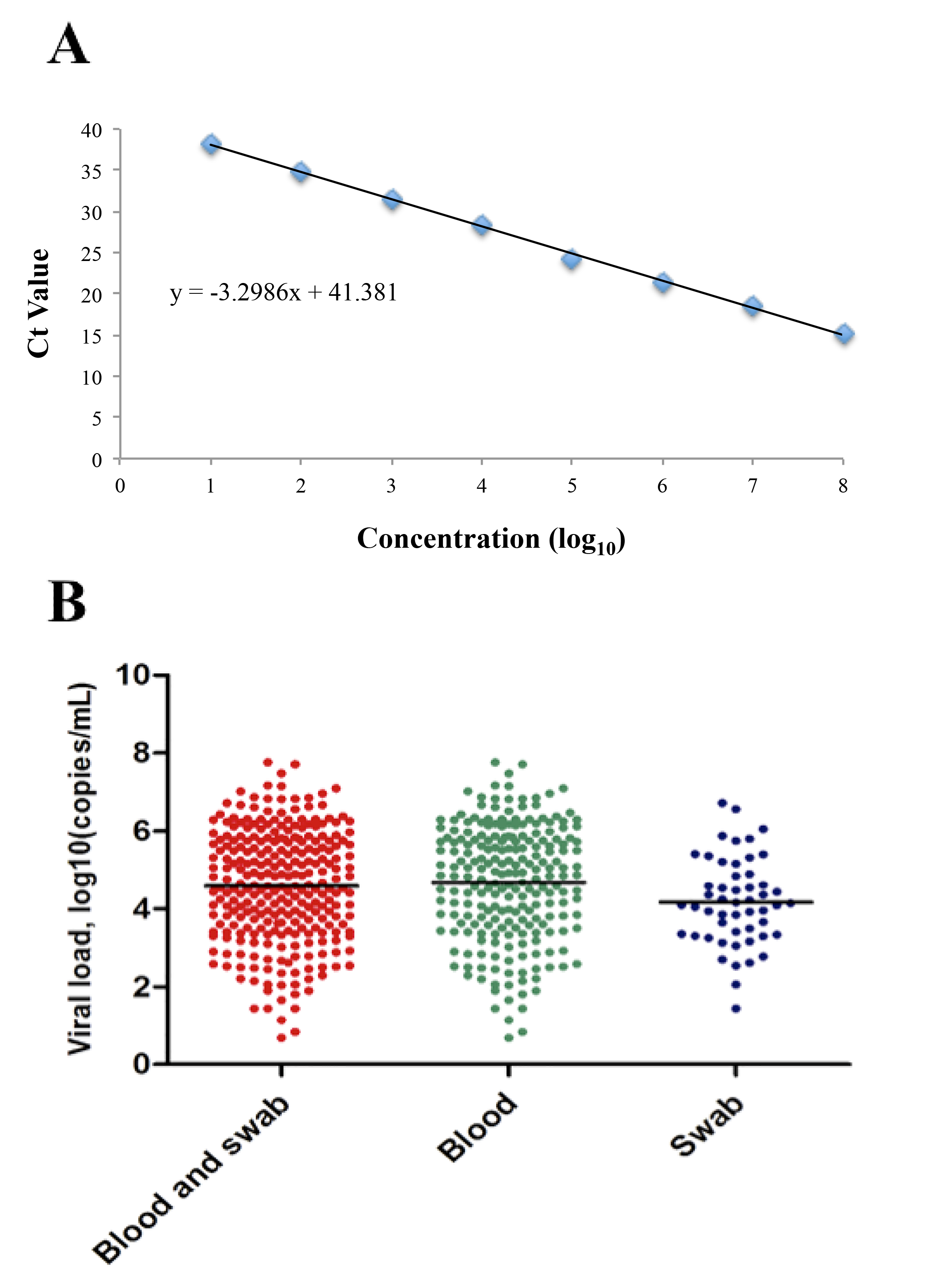


(A) Real-time PCR results obtained with a standard curve. Serial dilutions of plasmid DNA with concentrations ranging from 10^8^ to 10^1^ molecules/μL were detected by real-time PCR. The Ct values were plotted against the concentrations of the standards. (B) Viral loads of clinical samples calculated according to the standard curve.

### Figure S3 Distribution of Ct values and threshold times


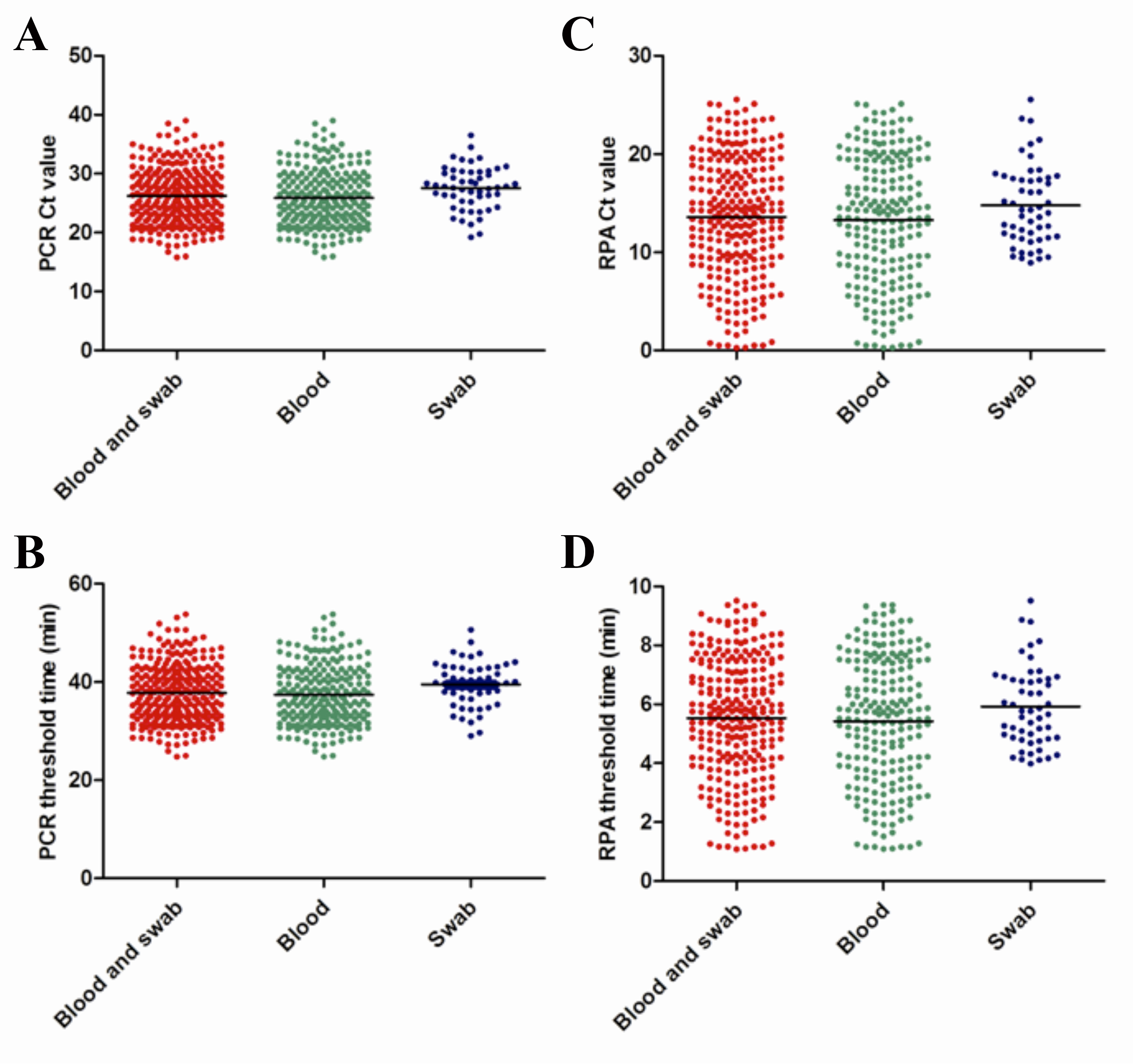


Distribution of the Ct values (A and C) and threshold times (B and D) of all the positive samples by RT-PCR (A, B) and EBOV-RPA (C, D)

### Figure S4 ROC curve of the EBOV-RPA assay for EVD


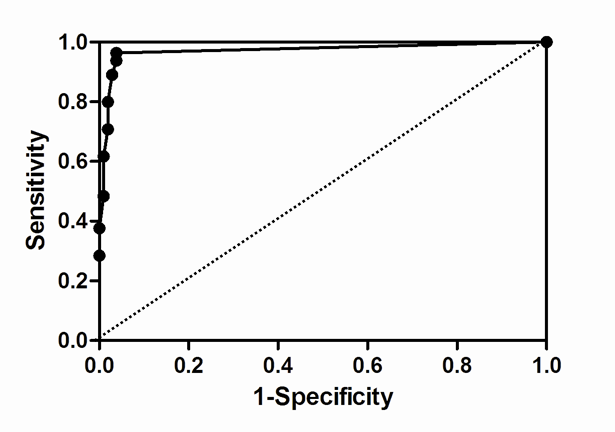


Sensitivity and specificity was calculated for each cutoff value from 10 to 26 (Ct values), and values for 1-specificity was plotted against the corresponding sensitivities to generate the ROC curve. The AUC was calculated based on the curve.

## Tables

### Table S1 Primers and probe used in the present study

| Name^a^ | Sequence (5' to 3')^b^ |
| --- | --- |
| EBO-RPA-F | TAGTAGGAGAAAAGGCTTGCCTTGAGAAGG |
| EBO-RPA-R | TGTTCGCATCAAACGGAAAATCACCATCATG |
| EBO-RPA-P | TCAAGTACATGCAGAGCAAGGACTGATACAAT(FAM)-(THF)-T(BHQ1)-CCAACAGCTTGGCAAT-(P) |

^a^ F or R, forward or reverse primers; P, exo-probe.

^b^ T(BHQ1), thymidine nucleotide carrying Black Hole Quencher 1; THF, tetrahydrofuran spacer; T(FAM), thymidine nucleotide carrying fluorescein; P, 3′phosphate to block elongation.

### Table S2 Effect of sample treatment on amplification

| **Sample treatment** | | **Dilution of treated samples^b^** | | | | | |
| --- | --- | --- | --- | --- | --- | --- | --- |
|  |  | **ND^a^** | **1: 2** | **1: 4** | **1: 8** | **1: 16** | **1: 32** |
| Sample/AVL | 2:1 | - | - | - | + | + | + |
|  | 1:1 | - | - | - | + | + | + |
|  | 1:2 | - | - | - | - | + | + |
|  | 1:4 | - | - | - | - | + | + |
| Sample/Trizol | 2:1 | - | - | + | + | + | + |
|  | 1:1 | - | - | - | + | + | + |
|  | 1:2 | - | - | - | + | + | + |
|  | 1:4 | - | - | - | - | + | + |
| Sample/Sample solution | 2:1 | + | + | + | + | + | + |
|  | 1:1 | + | + | + | + | + | + |
|  | 1:2 | + | + | + | + | + | + |
|  | 1:4 | + | + | + | + | + | + |

^a^ Not diluted

^b^ +, positive; −, negative.

### Table S3 Characteristics of CT values and threshold times observed in the RT-PCR and RPA assays

| Characteristics | | Minimum | Maximum | Mean | Medium | Range | 95% percentile | p value |
| --- | --- | --- | --- | --- | --- | --- | --- | --- |
| Ct values | | | | | | | | |
| RPA Ct values | 0.28 | | 25.56 | 13.58 | 13.9 | 25.28 | 23.31 | - |
| RT-PCR Ct values | 15.81 | | 39 | 26.22 | 26.21 | 23.19 | 34.28 |
| Threshold time | | | | | | | |  |
| RPA threshold time (min) | | 1.09 | 8.77 | 5.53 | 5.64 | 8.43 | 8.77 | 0.000 |
| RT-PCR threshold time (min) | | 24.76 | 53.75 | 37.78 | 37.76 | 28.99 | 47.84 |

### Table S4 Ct value distribution of the RT-PCR positive samples

| Ct range | Number | Percent |
| --- | --- | --- |
| <26 | 134 | 49.45% |
| 26–34 | 121 | 44.65% |
| >34 | 16 | 5.90% |
| 34–36 | 10 | 3.69% |
| 36–40 | 6 | 2.21% |

### Table S5 Detection results of blinded samples from External Quality Assessment (EQA)

| Sample ID | Sample Type | RPA (minutes) | GP | NP | In-house^a^ |
| --- | --- | --- | --- | --- | --- |
| EQA01 | Inactivated virus | NA | NA | NA | NA |
| EQA02 | Inactivated virus | 7.33 | 31.09 | 33.66 | 31.04 |
| EQA03 | Inactivated virus | 4.00 | 23.65 | 25.97 | 23.4 |
| EQA04 | Inactivated virus | NA | NA | NA | NA |
| EQA05 | Inactivated virus | 15.33 | 35.1 | 35.08 | 34.25 |
| EQA06 | Viral RNA | 5.68 | 26.9 | 29.13 | 26.49 |
| EQA07 | Viral RNA | NA | NA | NA | NA |
| EQA08 | Viral RNA | NA | NA | NA | NA |
| EQA09 | Viral RNA | NA | NA | NA | NA |
| EQA10 | Viral RNA | 13.24 | 33.81 | 33.61 | 34.38 |
| Weak positive control |  | 7.00 | 30.31 | 32.19 | 29.76 |
| Strong positive Control |  | 4.67 | 21.54 | 23.38 | 21.25 |
| Negative Control |  | NA | NA | NA | NA |

^a^ Assay developed as recommended by the WHO. As determined by the WHO EQA group, the results were 100% correct.
